# The magnitude of influence of personal and professional factors on the career choices of medical students

**DOI:** 10.25122/jml-2023-0453

**Published:** 2024-04

**Authors:** Emad Salawati, Ranya Ghamri, Ashraf Amir, Mohammed Alsubaie, Renad Abualsaud, Sarah Bahowarth, Lina Abdulrahman, Anas Alyazidi

**Affiliations:** 1 Department of Family Medicine, Faculty of Medicine, King Abdulaziz University, Jeddah, Saudi Arabia; 2 Department of Family Medicine, International Medical Center, Jeddah, Saudi Arabia; 3 Faculty of Medicine, King Abdulaziz University, Jeddah, Saudi Arabia

**Keywords:** medical student, future specialty, career intention, public policy, Saudi Arabia

## Abstract

Medical students face uncertainty in choosing their future careers, which is influenced by personal and professional factors and can have a direct impact on the healthcare system and society. In this study, we aimed to analyze the magnitude of influence of personal and professional factors on students’ choices of a future specialty, among medical students in Saudi Arabia and other Arab countries. This cross-sectional exploratory study used cluster sampling and collected demographic data, influencing factors, preferred specialty, and engagement level, using a 20-item questionnaire. The study included 1,105 students, of which 60.5% were women. Income was the most influential factor for both sexes (68.5%) and was statistically significant for men (*P* < 0.0001), with personal experience and type of patients served being more important for women. Many students (42.6%) were undecided about their future specialty. However, general surgery was the most preferred specialty, followed by internal medicine (10.9%) and obstetrics and gynecology (5.5%). Women had a significantly higher mean personal score than men, indicating a preference for surgery (3.07 ± 2.2 vs. 2.78 ± 2.1; *P* = 0.029). In this study, we found that income significantly influenced medical students’ choices of specialty, with surgery being the most common, and that many students are undecided about their specialty, needing career counseling and mentoring programs.

## INTRODUCTION

Medicine is a unique discipline characterized by uncertainty and critical decision-making, requiring consideration of different values and opinions [1]. This uncertainty and need for critical decisions can emerge as early as the first few years of medical school, when students begin contemplating their future career paths, often finding that their initial preferences are rarely fulfilled [2–4]. These decisions are challenging and influenced by numerous factors, including financial considerations, personal interests, personality traits, academic and educational performance, demographic characteristics, and cultural and societal values [5].

The scarcity of certain medical specialties and the unequal distribution of physicians are significant global healthcare issues [6]. Furthermore, imbalanced career choices among medical students and delays in their decision-making may also contribute to these problems. For example, evidence suggests that countries with strong primary care achieve better health outcomes and lower costs compared to countries with poor primary care [7]. However, it was consistently found that family medicine was one of the least popular specialties among medical students [8–10]. These issues are further complicated by a growing need for physicians, driven by factors such as aging populations, the increasing number of consultations, and the management of patients with multiple comorbidities [11].

In Saudi Arabia, it is currently estimated that over 50% of the population is aged 25 years and younger, with approximately 14% of Saudis aged between 15 and 24 years [12]. However, government projections indicate that the proportion of the Saudi population aged over 50 years and over 80 years will rise to 25% and 4%, respectively [13]. This means selecting a future specialty by current Saudi medical students is crucial to ensure a sufficient medical workforce and a balanced development of the medical system, as emphasized in the literature [14–16]. Therefore, identifying and analyzing the factors influencing medical students’ career choices is essential, as these decisions affect health systems, community health, as well as the personal and professional life of physicians.

In this study, we aim to explore the factors influencing medical students’ choice of specialty in Saudi Arabia and other Arab countries. The results will help medical schools and policymakers in shaping medical education and workforce planning strategies, improving recruitment in the region, especially during times of workforce imbalances. In addition, this study will help identify current struggles and barriers faced by medical students.

## MATERIAL AND METHODS

### Study design and setting

Following the Strengthening the Reporting of Observational Studies in Epidemiology (STROBE) guideline [17], we conducted a cross-sectional exploratory study in April 2023 in Saudi Arabia and other Arab countries (i.e., Egypt, Sudan, Lebanon, Yemen, Kuwait, United Arab Emirates, Oman, Qatar, Jordan, Iraq, Tunisia, Algeria, Morocco, Palestine, Syria, Libya, and Somalia) to explore, assess, and analyze the magnitude of influence of personal and professional factors on medical students’ career choices. Data were collected using a validated, self-administered questionnaire created with Google Forms and distributed electronically. A total of 1,105 participants completed the questionnaire. After applying the inclusion criteria and removing duplicate and incomplete responses, 98% of the responses were included in the analysis.

### Population and sampling

Medical students were randomly recruited using a cluster sampling method. Their status as medical students was verified through the survey, and pre-med students and students of other colleges were excluded. The statistically appropriate sample size was calculated using Raosoft software, with a 95% confidence interval (CI) and <0.05 margin of error. To ensure appropriate representation of this large population, we exceeded the minimum number of required participants, which was set at 385 for each group. The applied equation was as follows:

*n =* (DEFF × Np(1 − p))/((d2/Z21 − α/2 × (N − 1) + p × (1 − p)) [18]

where, *n =* population size, p = prevalence, d = precision (desired margin of error), DEFF = design effect, and Z1−α/2 = 1.96 for a 95% confidence level.

Overall, given that we included a total of 1,105 participants, exceeding the minimum sample size required for a 99.9% confidence interval, which was 1,083 participants.

### Data collection tool

We used a 20-item self-administered and validated questionnaire. At the beginning of the questionnaire, a consent statement explicitly outlined the research purpose, provided information about the study, and included contact details. Participants acknowledged that their information would be used for research purposes and confirmed that they were part of the targeted population. The first section of the questionnaire collected participants’ demographic data, including sex, date of birth, nationality, religion, current place of study, current year of study, language of study, current grade point average (GPA), marital status, and four questions assessing family status and household income.

The second section contained multi-select multiple-choice questions to assess influencing factors divided into personal and professional factors, based on a questionnaire validated by Chew *et al*. [19], Grasreiner *et al*. [20], and Chang *et al*. [21]. During the validation process, a two-tiered analytic hierarchy process (AHP) questionnaire was constructed. AHP is a measurement theory that uses binary comparisons and expert judgments to derive priority metrics, ultimately determining the relative weight of each factor [22]. Following its initial development, Grasreiner *et al*. [20] consulted five multidisciplinary specialist physicians in different centers for a primary revision, followed by three rounds of preliminary surveys, which eventually led to the current form of the survey. To adapt it to our targeted population, we used the same questionnaire as Alyazidi *et al*. [23], maintaining its validation construct. In addition, these factors were adjusted into a Personal Factor Score (PEFS) and a Professional Factor Score (PRFS). The questionnaire was carefully revised to ensure that it met the study’s objectives. A pilot study was conducted to verify the clarity and accuracy of responses. The validity of the questionnaire was further reviewed by epidemiologists, statisticians, and public health specialists.

The third section assessed participants’ preferred specialty using single-select multiple-choice questions. The specialties included internal medicine (encompassing cardiology, gastroenterology, and infectious diseases), surgery (including general surgery, neurosurgery, vascular surgery, and urology), orthopedic surgery, plastic surgery, pediatrics, obstetrics and gynecology, neurology, ophthalmology, otorhinolaryngology, dermatology, emergency medicine, anesthesiology, psychiatry, family medicine, preventive medicine, public health, radiology, laboratory medicine, basic science, and an option for those still undecided. The classification of specialties was based on the study’s objectives.

The fourth and final section included ‘yes’ or ‘no’ questions assessing the level of engagement in the chosen specialties. This section included the following items: A) Attended a lecture or workshop related to their chosen specialty; B) Participated in elective clinical training related to their chosen specialty; C) Volunteered or enrolled in extracurricular or social activities related to their chosen specialty; D) Discussed the desired specialty with a practicing doctor in the field; E) Participated in any research activity related to their chosen specialty. These items were grouped into a Knowledge Factor Score (KNFS). The questionnaire was pre-tested among 60 students from the targeted population to ensure clarity, accuracy, and consistency. We assessed the pre-test group’s understanding and consistency in responding to the questionnaire.

### Statistical analysis

Statistical analyses were performed using SPSS v.21.0 for Windows (IBM Corp). For descriptive statistics, continuous variables were summarized using mean and s.d., whereas categorical variables were presented using numbers and percentages. The chi-squared test or Fisher’s exact test (when any cell’s expected count was lower than 5) was used to compare the influencing factors between sexes. Significant variables identified in the univariate analysis were further tested using multivariate logistic regression analysis to identify independent influencing factors, with results presented as odd ratios within a 95% CI. A *P* value of ≤0.05 was considered statistically significant.

## RESULTS

### Participant characteristics

The study included 1,105 students, 60.5% of which were women. The majority of participants (70.1%) were aged between 22 and 26 years, and 87.4% were Muslims. Regarding the current year of study, 46.7% were in their basic years, 43.6% were in their clinical years, and 9.7% were in their internship year. Most of the participants (73.8%) were first-generation medical students. The demographic characteristics of the participants are presented in Table 1.

**Table 1:** Demographic characteristics of the participants

Demographic characteristics	Women (*n* = 668)	Men (*n* = 437)	Total (*n* = 1,105)
**Age (years)**			
17–21	223 (33.4%)	50 (11.4%)	273 (24.7%)
22–26	426 (63.8%)	349 (79.9%)	775 (70.1%)
27–31	19 (2.8%)	38 (8.7%)	57 (5.2%)
**Nationality**			
Saudi	277 (41.5%)	158 (36.2%)	435 (39.4%)
Non-Saudi	391 (58.5%)	279 (63.8%)	670 (60.6%)
**Religion**			
Muslim	587 (87.9%)	379 (86.7%)	966 (87.4%)
Non-Muslim	81 (12.1%)	58 (13.3%)	139 (12.6%)
**Marital status**			
Single	646 (96.7%)	425 (97.2%)	1,071 (96.9%)
Married	22 (3.3%)	12 (2.7%)	34 (3.1%)
**Place of study**			
Saudi Arabia	297 (44.5%)	171 (39.1%)	468 (42.4%)
Other Arab countries	371 (55.5%)	266 (60.9%)	637 (57.6%)
**Language of study**			
English	578 (86.5%)	171 (39.1%)	468 (42.4%)
Arabic	90 (13.5%)	266 (60.9%)	637 (57.6%)
**Current year of study**			
Basic years	375 (56.1%)	141 (32.3%)	516 (46.7%)
Clinical years	247 (37.0%)	235 (53.8%)	482 (43.6%)
Internship	46 (6.9%)	61 (14.0%)	107 (9.7%)
**Current GPA**			
A or A+	307 (46%)	174 (39.8%)	481 (43.5%)
B or B+	255 (38.2%)	186 (42.6%)	441 (39.9%)
C or C+	25 (3.7%)	18 (4.1%)	43 (3.9%)
D or D+	81 (12.1%)	59 (13.5%)	140 (12.7%)
**First generation student**			
Yes	477 (71.4%)	339 (77.6%)	816 (73.8%)
No	191 (28.6%)	98 (22.4%)	289 (26.2%)

### Influencing factors and sex-related variations

The frequency of influencing factors according to participants’ sex is shown in Table 2. For professional factors, income was the most influential factor for both sexes (68.5%). However, it had a significantly greater influence on men than on women (82.2% vs. 59.6%; *P* < 0.0001). The second most influential factor was career prospects (65.3%), which also had a greater influence on men than women (70.5% vs. 62%; *P* = 0.004). Furthermore, men were significantly more affected by night calls. By contrast, women were more influenced by the lack of experts in their countries, the length and difficulty of the training period, and work-related hazards.

**Table 2:** Influencing factors

Influencing factors	Women (*n* = 668)	Men (*n* = 437)	Total (*n* = 1,105)	*P* value
**Professional factors**				
Income	398 (59.6%)	359 (82.2%)	757 (68.5%)	<0.000^1^*
Workload	229 (34.3%)	148 (33.9%)	377 (34.1%)	0.887
Career prospects	414 (62.0%)	308 (70.5%)	722 (65.3%)	0.00^4^*
Advice from practicing doctor	171 (25.6%)	120 (27.5%)	291 (26.3%)	0.492
Lack of experts in the country	221 (33.1%)	101 (23.1%)	322 (29.1%)	<0.0001^*^
Length and difficulty of training period	217 (32.5%)	112 (25.6%)	329 (29.8%)	0.015^*^
Very challenging nature of this field	180 (26.9%)	95 (21.7%)	275 (24.9%)	0.050
Work-related hazards	96 (14.4%)	42 (9.6%)	138 (12.5%)	0.019^*^
Continuous care and extent of patient contact	120 (18.0%)	67 (15.3%)	187 (16.9%)	0.254
No night calls	86 (12.9%)	81 (18.5%)	167 (15.1%)	0.010^*^
**Personal factors**				
Social prestige	156 (23.4%)	104 (23.9%)	260 (23.6%)	0 .859
Personal experience	209 (31.3%)	100 (22.9%)	309 (28.0%)	0.002^*^
Number and type of patients served	141 (21.2%)	53 (12.1%)	194 (17.6%)	<0.0001^*^
Advice from parents/family	197 (29.5%)	136 (31.1%)	333 (30.1%)	0.564
Advice from friends/seniors	162 (24.3%)	73 (16.7%)	235 (21.3%)	0.003^*^
Less working hours, to spend time with family	161 (24.1%)	110 (25.2%)	272 (24.5%)	0.686
Less work pressure and better quality of life	187 (28.1%)	160 (36.6%)	347 (31.5%)	0.003^*^
Possession of competency needed	322 (48.2%)	209 (47.8%)	531 (48.1%)	0.902
Academic or teaching opportunity	157 (23.5%)	63 (14.4%)	220 (19.9%)	<0.0001^*^
Participation in research	180 (26.9%)	91 (20.8%)	271 (24.5%)	0.021
Ability to migrate	188 (28.1%)	116 (26.5%)	304 (27.5%)	0.561

* Statistically significant. The chi-squared test was used to compare between sexes.

Regarding personal factors, women were more influenced by their own personal experiences, the number and type of patients served, advice from friends or seniors, and academic or teaching opportunities. Men, on the other hand, were more affected by less work pressure and a better quality of life.

In the multivariate logistic regression analysis, which included all significant influencing factors identified in the univariate analysis, independent influencers by sex were determined. These included income (*P* < 0.0001; OR = 0.338; 95% CI, 0.247–0.463), absence of night calls (*P* = 0.036; OR = 0.651; 95% CI, 0.436–0.973), personal experience (*P* = 0.016; OR = 1.46; 95% CI, 1.072–1.99), advice from friends or seniors (*P* = 0.045; OR = 1.429; 95% CI, 1.009–2.025), and academic or teaching opportunities (*P* = 0.049; OR = 1.442; 95% CI, 1.001–2.077) (Table 3).

**Table 3:** Results of the multivariate logistic regression analysis of the influencing factors

Factor	*P* value	Odds ratio	95% CI	
			Lower	Upper
Income	<0.0001^*^	0.338	0.247	0.463
Career prospects	0.214	0.831	0.621	1.113
Lack of experts in the country	0.052	1.357	0.998	1.844
Length and difficulty of training period	0.165	1.243	0.914	1.690
Work-related hazards	0.588	1.130	0.727	1.754
No night calls	0.036^*^	0.651	0.436	0.973
Personal experience	0.016^*^	1.460	1.072	1.990
Number and type of patients served	0.053	1.454	0.995	2.125
Advice from friends/seniors	0.045^*^	1.429	1.009	2.025
Less work pressure and better quality of life	0.211	0.818	0.598	1.121
Academic or teaching opportunity	0.049^*^	1.442	1.001	2.077

* Statistically significant. Multivariate logistic regression was used to identify independent influencing factors.

### Specialty preference

**Figure 1:**
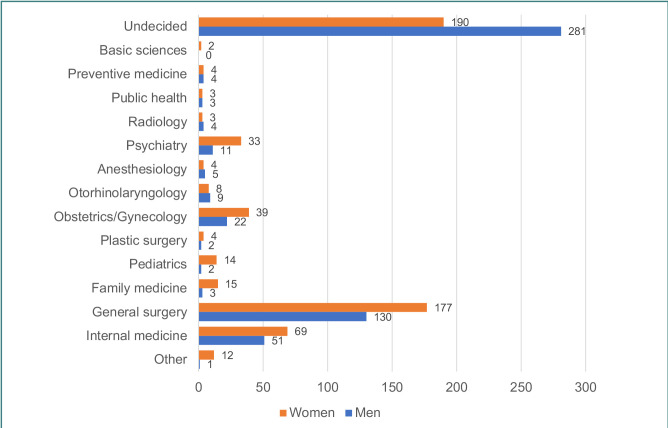
Specialty preferences. Data presented as frequency.

Figure 1 presents specialty preferences based on participants’ sex. The majority of students (42.6%) had not yet decided on their future specialty. Among those who had chosen a specialty, general surgery was the most preferred (27.8%), followed by internal medicine (10.9%) and obstetrics and gynecology (5.5%). There were no statistically significant differences between sexes in regard to specialty preferences.

### Professional, personal, and knowledge scores

Comparisons of the professional, personal, and knowledge scores based on demographic data are presented in Table 4. Women had significantly higher mean personal scores than men (3.07 ± 2.2 vs. 2.78 ± 2.1; *P* = 0.029). In addition, non-Muslims had much higher mean professional and personal score than Muslims. Furthermore, first-generation medical students had considerably higher mean professional and knowledge scores compared to continuing-generation medical students.

**Table 4:** Comparison of mean professional, personal, and knowledge scores

Demographic characteristics	Mean PEFS (0–11)	Mean PRFS (0–10)	Mean KNFS (0–5)
**Age (years)**			
17–21	2.79 ± 2.1	2.68 ± 1.9	1.74 ± 1.5
22–26	3.03 ± 2.2	3.42 ± 1.9	1.88 ± 1.4
27–31	2.79 ± 1.4	3.16 ± 1.5	1.67 ± 1.5
**Sex**			
Male	2.78 ± 2.1	3.28 ± 1.8	1.73 ± 1.4
Female	3.07 ± 2.2	3.19 ± 2.0	1.90 ± 1.5
**Nationality**			
Saudi	2.98 ± 2.2	3.33 ± 2.0	1.95 ± 1.4
Non-Saudi	2.94 ± 2.1	3.16 ± 1.9	1.76 ± 1.5
**Religion**			
Muslim	2.87 ± 2.1	3.09 ± 1.9	1.84 ± 1.4
Non-Muslim	3.58 ± 2.4	4.16 ± 2.2	1.83 ± 1.5
**Marital status**			
Single	2.97 ± 2.1	3.23 ± 1.9	1.86 ± 1.5
Married	2.74 ± 1.7	3.44 ± 2.0	1.21 ± 1.1
**Current year of study**			
Basic years	2.70 ± 2.1	2.89 ± 1.9	1.78 ± 1.4
Clinical years	3.28 ± 2.2	3.48 ± 2.0	1.88 ± 1.5
Internship	2.79 ± 1.7	3.72 ± 1.5	1.89 ± 1.2
**Current GPA**			
A or A+	2.94 ± 2.3	3.33 ± 2.1	1.74 ± 1.3
B or B+	3.02 ± 2.1	3.17 ± 1.8	1.97 ± 1.7
C or C+	2.67 ± 1.8	2.42 ± 1.5	1.65 ± 0.8
D or D+	2.92 ± 1.8	3.29 ± 1.8	1.81 ± 1.4
**First generation student**			
Yes	2.95 ± 2.0	3.30 ± 1.9	1.95 ± 1.5
No	2.97 ± 2.4	3.02 ± 2.0	1.51 ± 1.3

Data presented as mean ± s.d.

## DISCUSSION

Our study focused on the factors that influence medical students’ decisions regarding their prospective career specialties. The findings demonstrate that a variety of factors affect these decisions. Some of them are non-modifiable, such as age and sex, whereas other are slow to change, like the perceived characteristics of a specialty. In addition, there are modifiable factors, such as the amount of clinical exposure and obtaining an expert opinions, which can considerably affect career choices. Notably, we found a growing interest in surgical specialties, with income being an influencing factor for both men and women. Our findings also indicate that a substantial proportion of students remain undecided about their preferred future specialty.

### Personal data and influencing factors

In terms of the current academic year, 43.6% of students were in their clinical years and 46.7% were in their primary years. The majority of participants were first-generation medical students. Because an effective physician workforce policy requires a thorough understanding of the factors influencing specialty choice, we aimed to provide a comprehensive overview of the data from our analysis rather than focusing on a single specialty. We also examined these factors to identify knowledge gaps and potential areas for further study.

Our research revealed that general surgery was the most favored specialty (27.8%), followed by internal medicine (10.9%). Surgery was the most popular specialty among both male and female students. It is particularly noteworthy that there is an increasing prevalence of women choosing a career in surgery, especially in a conservative eastern society. This trend mirrors the findings of another study in Saudi Arabia, in which students chose general surgery as their major, followed by pediatrics and internal medicine [24]. Another nationwide study concluded that surgery was the preferred specialty among male students, followed by internal medicine and orthopedics [25]. Similarly, another study found that general surgery was the most preferred career specialization among the evaluated cohort of students [26]. Factors such as mentorship, intellectual stimulation, the fulfilling nature of surgery, and exposure to various specialties likely have a beneficial effect on women choosing this field.

In contrast to women, men showed a preference for seeking prestige and higher income [27]. Interestingly, income had the most significant impact for both sexes, with male students particularly focused on pursuing careers with higher earnings. A survey conducted among medical students in Saudi Arabia also highlighted the significant role of income in students’ career decisions [28]. Similarly, among male students in Pakistan, the expected salary, and a positive work environment were key factors influencing career choices [29]. However, a Nigerian study found that financial return had no impact on specialty preference [30]. This may be attributed to the traditional role of men as providers for their families, making income a more significant consideration for them compared to women.

In addition, our analysis revealed that 43% of students had not yet chosen their specialty. This finding aligns with a previous survey of college students in Iraq, which reported that 19% of students were undecided [31]. In another study conducted in Botswana, 10.3% of medical students were still deciding at the time of the research [32]. Insufficient exposure to different specialties during the clinical years may also be a barrier, as students may not fully understand the various aspects of each field. It is possible that a substantial portion of the respondents in our survey did not receive career counseling, contributing to their indecision about their preferred specialty. These students may ultimately be influenced by personal interests or peer and family pressure in their specialty choice. It is important to note that although our survey limited respondents to selecting only one option for career considerations, individuals typically have multiple options to consider in real-life circumstances.

### Preferred specialties

We found a lower percentage (57%) of students who had decided on their career choice compared to previous local studies, which reported 67% and 80%, and international studies among Greek and Botswanan medical students, in which 97% and 90% of students had decided, respectively [25,32–34]. Surgery, internal medicine and obstetrics and gynecology were among the most preferred specialties, consistent with findings reported by Khader *et al*. [8], Avgerinos *et al*. [34], and Mariolis *et al*. [35]. Although other studies have found significant differences between men and women regarding specialty choice [33,36,37], we found no significant differences between sexes. The most preferred specialty among both male (30%) and female (26%) medical students who were not undecided was general surgery. It is noteworthy that general surgery was the most preferred specialty among decided female medical students, contrary to multiple studies that reported that women were less likely to prefer surgical disciplines [8,38–40]. Multiple studies found family medicine to be among the least popular specialty preferences [8,33,41]. Similarly, our study found family medicine to have a low preference, being preferred by only three male students (0.6%); however, it was the fifth most preferred specialty (2.2%) among female students. Family medicine was also more preferred than pediatrics by both sexes (0.4% for men and 2.2% for women), a specialty consistently found to be among the top four desirable choices [32,42,43]. The low level of interest in anesthesiology, public health, and basic medical sciences is consistent with other studies [33,42,44]. Regarding the mean PRFS, PEFS, and KNFS scores, a trend was observed among participants. Generally, female students had a higher mean PEFS, echoing studies suggesting that women in Eastern societies are more likely to be influenced by family-related factors [45]. An increase in PRFS was also seen as participants advanced in their college years.

### Study limitations

Sample bias, as well as unequal number of participants and variability in their demographic characteristics, could potentially be limitations in the study. However, these limitations were addressed by substantially increasing the sample size and ensuring an appropriate representation across different demographics.

## CONCLUSION

This study investigated the factors influencing medical students’ decisions about their chosen specialties in Saudi Arabia and other Arab countries. The most commonly chosen specialty was surgery, with income being the most important determining factor for both men and women. However, a large percentage of students had not yet decided on their specialty. As a result, career counseling and mentoring programs are necessary to support these students in making informed decisions about their future careers.

## Data Availability

All data generated or analyzed during this study are included in the article. The datasets used and/or analyzed are available from the corresponding author upon reasonable request.
